# A Systematic Review and Meta-Analysis of Measurement Feedback Systems in Treatment for Common Mental Health Disorders

**DOI:** 10.1007/s10488-022-01236-9

**Published:** 2022-11-25

**Authors:** Kristian Rognstad, Tore Wentzel-Larsen, Simon-Peter Neumer, John Kjøbli

**Affiliations:** 1 Regional Center for Child and Adolescent Mental Health, Eastern and Southern Norway, Oslo, Norway; 2 Department of Psychology, University of Oslo, Oslo, Norway; 3 Norwegian Centre for Violence and Traumatic Stress Studies, Oslo, Norway; 4 Regional Centre for Child and Youth Mental Health and Child Welfare, UIT The Arctic University of Norway, Tromsø, Norway; 5 Department of Education, University of Oslo, Oslo, Norway

**Keywords:** Measurement feedback systems, Outcome monitoring, Psychotherapy, Meta-analysis, Three-level analysis

## Abstract

To investigate the effects of measurement feedback systems (MFSs) in therapy on mental health outcomes through a literature review and meta-analysis. Using a three-level modeling approach, we conducted a meta-analysis of all effect sizes from randomized controlled studies of MFSs used in the treatment of common mental health disorders. Eighty-two effect sizes were extracted from the thirty-one included studies. Analyses were performed to consider the post-treatment effects of the MFS-assisted treatment compared to treatment as usual. A separate analysis was done for the subgroup “not-on-track” patients as it is theorized that MFSs will be clinically useful because they make therapists aware of patients who fail to progress. MFSs had a significant effect on mental health outcomes (*d* = 0.14, 95% CI [0.082–0.206], p < .001). Further analysis found a larger effect in patients identified as less respondent to therapy, the “not-on-track” group (*d* = 0.29, 95% CI [0.114, 0.464], p = .003). Moderation analyses indicated that the type of outcome measurement and type of feedback system used, and whether it was used for a child and youth or adult population, influenced effect sizes. MFSs seem to have a small positive effect on treatment outcomes. The effects seem to be larger for “not-on-track” patients, the group of patients that would usually not benefit much from treatment.

**Supplementary Information:**

The online version contains supplementary material available at 10.1007/s10488-022-01236-9.

Although therapy for mental health disorders is helpful for a majority of patients, for a large share of patients, therapy will be ineffective (Wolpert, 2016). About 5–10% of participants in clinical trials end treatment worse off than they began (Lambert & Shimokawa, 2011; Hannan et al. 2005). Even more patients have no discernable effects on symptoms from receiving psychotherapy (Cuijpers et al., 2019). Estimates indicate that among children and youth, 10–20% of patients end therapy with more symptoms than they initially reported (Brière et al., 2016; Warren et al., 2010). Such high rates of deterioration and failure to alleviate patient distress should be taken seriously.

Measurement feedback systems (MFSs) have been developed and tested to address this problem by providing therapists, patients, and other stakeholders with additional indicators of changes in patients’ symptoms, well-being, and functioning throughout the treatment period. MFSs that continuously provide systematic information about the client are assumed to be beneficial for the quality of therapy and have become more common in recent years as primary studies have found that MFSs can improve therapy outcomes (e.g. Amble et al., 2015; Bickman et al., 2011). Ideally, MFSs should guide therapeutic interventions and case conceptualization towards the client’s perspective (Cooper et al., 2021). Moreover, MFSs could result in better resource allocation in cases where patients whose symptoms do not improve get more tailored treatment and patients with positive development end treatment earlier (e.g., Lambert & Shimokawa, 2011; Lambert et al., 2001).

MFSs with standardized indicators of patient improvement may help therapists be aware of changes, or lack thereof, and more responsive to patients’ needs. Without such help, therapists can be poor at predicting which of their patients will deteriorate (Hannan et al., 2005) and they do not necessarily detect deterioration in cases with distinct escalation in symptoms (Chapman et al., 2012; Hatfield et al., 2010). There is also a lack of agreement between therapists and their clients regarding how well clients are doing and progressing (Bar-Kalifa et al., 2016). Congruence between client and therapist assessments of client symptoms and functioning is predictive of better outcomes. By assisting therapists with independent, standardized measures, MFSs can support decisions in therapy that are related to patients’ experiences and the fluctuations in the symptoms and less influenced by therapists’ framing of questions or awareness of change.

In addition, therapists often misjudge clients’ perceptions of the therapeutic relationship. Studies indicate that there is a lack of agreement between therapist and patient ratings of therapist empathy and therapeutic relationship measures (e.g., Chapman et al., 2012; Free et al., 1985). More similar alliance ratings from therapists and patients are associated with better clinical outcomes (Laws et al., 2017). Thus, many MFSs include measures of therapeutic alliance to help identify early ruptures in the patient-therapist relationship.

In MFSs permitting clients to review the data, it may also help with their self-reflection and sense of ownership of the therapeutic process. An MFS can enhance collaboration between service provider and service user (Tollefsen et al., 2020a, 2020b) as well as strengthen patients’ sense of control over their life circumstances and mental health (Tollefsen et al., 2020b). By presenting patients with the MFS output, they may have a better understanding of what is needed for change and it could lead to greater engagement. Accordingly, one study indicated that feedback to patients in addition to therapists had positive consequences for clinical outcomes (De Jong et al., 2014).Previous reviews have concluded that MFSs are associated with faster and better diagnostic procedures, better communication between patient and therapist (Carlier et al., 2012), and superior treatment outcomes compared to treatment as usual (TAU) in the majority of studies (Gondek et al., 2016) and improve short-term mental health outcomes (Cohen’s *d* = 0.10, Knaup et al., 2009). Other reviews have focused exclusively on the most frequently used MFSs, namely the Outcome Questionnaire (OQ) and the Partners for Change Outcome Management System (PCOMS), finding positive effects on clinical outcomes for both systems when provided in combination with TAU. Shimokawa et al. (2010) combined data from six OQ studies in a meta-analysis and reported an overall effect of Hedges’ *g* = 0.28 (*p* = 0.003) when OQ is added to TAU. Lambert et al. (2018) conducted separate meta-analyses for 9 PCOMS and 15 OQ studies, finding effect sizes on outcome compared to TAU of *d* = 0.14 and 0.40, respectively. A more recent study included 18 PCOMS studies (14 RCTs and 4 non-randomized studies) and found a small effect of *g* = 0.27 (*p* < 0.001) favoring PCOMS over control conditions (Østergård et al., 2020).

In the most comprehensive review of randomized controlled trials (RCTs) testing effects of MFSs to date, Kendrick et al. (2016) identified 17 studies in adult populations. Their meta-analysis of 12 studies using the OQ or the PCOMS showed no significant difference in treatment outcomes between MFS conditions and no-MFS conditions. Further, the authors noted that the identified studies were of low quality and the meta-analysis was based on few studies, which raises questions about the reliability of the results, and asserted that “further research is very likely to have an important impact on our confidence in the estimate of effect and is likely to change the estimate” (p. 30). Similarly, Bergman et al. (2018) conducted a Cochrane review of MFSs in child and youth psychotherapy but found a limited number of studies (k = 6) and were unable to perform a meta-analysis. In the last few years, several more primary studies on MFSs have been conducted. More recently, de Jong et al. (2021) included both RCTs and non-randomized studies in a meta-analysis and found a small overall effect of MFSs on symptom reduction (Cohen’s *d* = 0.15). The current study adds to the searches from Kendrick et al. (2016) and Bergman et al. (2018) and provides an up-to-date meta-analysis on the effects of MFSs in RCTs. While we were able to include more studies than Kendrick et al. (2016) and Bergman et al. (2018), we only included RCTs, in contrast to De Jong et al. (2021), to increase the certainty that the effects observed in the studies were caused by the MFSs as non-randomized studies may yield biased intervention effect estimates (Rossi et al., 2018).

The MFS literature often differentiates between on-track (ON) and not-on-track (NOT) patients. ON patients are those with a pattern of change in outcome measures that indicate improvement during therapy, while NOT patients have slopes of change that indicate no symptom change or negative change. Potential effects of MFSs likely come from the increased possibility that therapists will detect NOT patients early in treatment. This assumption has been strengthened by several studies showing that MFSs have particularly beneficial effects on NOT patients (e.g., Lambert et al., 2001; Whipple et al., 2003). Similar effects have been found in meta-analyses and reviews where overall effects are greater in the NOT groups (Gondek et al., 2016; Kendrick et al., 2017; Shimokawa et al., 2010). Therefore, in line with previous meta-analyses, we calculated an overall effect estimate for NOT patients in addition to the main overall estimate.

MFSs in their most elementary form merely provide therapists with routine information that the patient shares. Some feedback systems have additional functions, including comparing responses to norms or slopes from patients who respond well to treatment and alerting therapists when patients are diverging from this slope or when items indicate risk behavior (e.g., suicidal ideation, use of drugs). Other systems provide problem-solving recommendations or clinical support tools (CSTs) with guidelines for therapeutic responses to patients based on the problems they are reporting (e.g., Harmon et al., 2007; Slade et al., 2008; Whipple et al., 2003). There is one existing meta-analysis, albeit with only two studies with both MFSs and CSTs, that uses CSTs as a moderator (Shimokawa et al., 2010). The moderator effects of CSTs were insignificant in Shimokawa et al. (2010), but the authors noted a small therapeutic gain for NOT patients. In the current study, we examined CSTs as a moderator as we expected this to now be more frequent in the literature.

Two of the most common MFSs used in the research literature are OQ and PCOMS. To consider whether these more established types of MFS have a greater effect on treatment outcomes than other types of MFS, we used MFS type as a moderator in the meta-analysis.

In summary, the literature is inconclusive as to whether MFSs can be expected to have positive effects on common mental health disorders. However, during the last few years, a substantial number of RCTs examining the effects of MFSs have been added to the research literature. Although the reviews by Kendrick et al. (2016) and Bergman et al. (2018) were published quite recently, our impression from the field and number of preregistered trials (e.g., in www.isrctn.com and ClinicalTrials.gov) is that a new search would result in a substantial increase in the number of studies and concomitantly increased certainty in the results of a meta-analysis.

The use of improved statistical methods is another source of increased certainty from a systematic synthesis. In contrast to previous meta-analyses, and to use all available data from the individual studies, a three-level analysis was conducted to make use of all calculable effect sizes (Assink & Wibbelink, 2016). In traditional meta-analytic approaches, all the effect sizes must be independent. Dependency of effect sizes represents an overlap in information and could lead to overconfidence in the meta-analytic results. Thus, to achieve independence, typically only one effect size was included from each study (e.g., Lipsey & Wilson, 2001). This, in turn, is questionable for other reasons, as extracting one effect size from each study assumes homogeneity of effect sizes within studies. By applying a three-level model to meta-analysis, the biases associated with independence can be mitigated as one can allow effect sizes to vary between participants (level 1), outcomes (level 2), and studies (level 3) (Assink & Wibbelink, 2016).

We aimed to (1) examine whether MFSs have effects on outcomes from treatment of common mental health disorders; (2) examine the overall effect for patients identified as “not-on-track”; and (3) explore candidate moderators based on prior meta-analyses (i.e., Kendrick et al., 2016, Shimokawa et al., 2010) and frequent variation in the field, including length of treatment, type of outcome measure, type of MFS, presence or absence of CSTs or similar modules, treatment context, and child and youth or adult population, and recipient of the feedback (therapist only or therapist and patient).

## Methods

We preregistered the review in PROSPERO (Code CRD42021240379) and followed the preferred reporting items for systematic reviews and meta-analyses (PRISMA) guidelines (Page et al., 2021).

### Eligibility Criteria for the Systematic Review

We planned to include randomized controlled trials, both cluster-randomized and randomized at the level of individual participants. The control condition should be similar to the active condition except for the MFS and CST tested. We included only RCTs, which means that our meta-analysis has a narrower and more stringent scope than that of De Jong et al. (2021). In addition, we only included “common mental health disorders,” defined here as depression, mixed anxiety and depression, and specific anxiety disorders, such as generalized anxiety disorder (GAD), phobias, obsessive–compulsive disorder (OCD), panic disorder, and post-traumatic stress disorder (PTSD). We excluded studies with more than 10% of the sample in drug/alcohol treatment or with dementia, learning disorders, or psychosis. We deemed studies of these patient populations to be too different in terms of both what information is considered clinically relevant and the patient’s self-awareness or ability to report. We excluded studies of MFSs in group therapy or couples’ therapy as we suspect the potential for changing the course of therapy based on feedback is different in those contexts. Although interesting in these fields as well, both the self-reporting and the potential for use might make the feedback loop different in the excluded populations.

In the current review, we used the same inclusion and exclusion criteria as the aforementioned Cochrane reviews (Kendrick et al., 2016; Bergman et al., 2018), including both studies with child and with adult populations. We updated the searches from these reviews to include all new studies of measurement feedback systems that monitor client progression and report to therapists.

#### Inclusion

##### Design

Randomized controlled trials, both cluster-randomized and randomized at the level of participants.

##### Participants

Participants in treatment with common mental health disorders, the majority of participants having such a diagnosis or clinical assessments indicating such a problem. Any age group was included.

##### Intervention

Patient outcome data given to therapists, patients, or both, on a regular basis for the duration of therapy.

##### Context

Any primary care, out- or inpatient therapy, multidisciplinary mental health care, psychological therapies.

##### Subset Data

Include studies where subsets of the data may qualify (fulfill criteria 1–4), e.g., three-armed RCTs, a portion of participants are relevant and can be extracted.

#### Exclusion

##### Design

Excluded any non-randomized design, including comparison of assumed similar groups treated at different time periods, or benchmark studies. Excluded any studies comparing MFS to other treatment options besides TAU. Excluded studies where the intervention arm also included other manual-based or otherwise defined interventions not available to both the intervention and control groups.

##### Participants

Excluded studies with more than 10% of the sample in therapy for drug/alcohol treatment, dementia, learning disorders, or psychosis, as well as studies with more than 10% of the sample with eating disorders.

##### Context

Excluded studies of couples’ therapy or group therapy.

### Inclusion and Exclusion Criteria for the Meta-Analysis

All studies that reported data, either in the articles or through email correspondence, allowing for a calculation of effect size (Cohen’s *d*) and the variance of effect size were included in the meta-analysis.

### Information Sources and Search

All articles identified in Kendrick et al. (2016) were added to the full-text screening phase of the review. We assumed that articles published before this search were included. We allowed for a potential lag in publications and database registrations, and thus set our search from 2014 although the last search update in Kendrick et al. (2016) was conducted on May 18, 2015. The Cochrane Central Register of Controlled Trials (CENTRAL), American Psychology Associations’ PsychInfo, Ovid MEDLINE and Epub Ahead of Print, In-Process, In-Data-Review & Other Non-Indexed Citations, Daily and Versions and Excerpta Medica Database (EMBASE) were searched for articles published from 2014. Search terms are available in additional resources. The last search was done on May 12, 2022.

Similarly, all articles from Bergman et al. (2018) were added to the full-text screening. New searches in the Cochrane Central Register of Controlled Trials (CENTRAL), American Psychology Associations’ PsychInfo, Ovid MEDLINE(R), Epub Ahead of Print, In-Process, In-Data-Review & Other Non-Indexed Citations, Daily and Versions, and Excerpta Medica Database (EMBASE) were performed to update the search from Bergman et al. (2018). Search terms are available in additional resources. The last search was done on May 5, 2022.

All references were added to the Covidence systematic review software (Veritas Health Innovation, Melbourne, Australia; available at www.covidence.org); two reviewers considered each abstract and full text and assessed the risk of bias (RoB). Abstracts and full texts were screened independently by each reviewer. When conflicts or uncertainty occurred within pairs of reviewers, full-text articles were obtained, and disagreement was discussed.

### Meta-analytic Data Extraction Process

Two reviewers independently extracted data. Meetings were held to reach agreement and produce a final dataset. The post-treatment measurements mean, standard deviations, and number of respondents were extracted from the identified articles. Reported effect sizes were extracted where mean or standard deviation was not reported.

In cases where post-treatment measurements were lacking, follow-up measurements were applied as an outcome. The measurements closest to average treatment length were used. In Rise et al. (2016), the measurement from six months after the start of treatment was used. If treatment length was not reported, the measures closest to six months after the start of treatment were included in the meta-analysis (6-month follow up in Kendrick et al., 2017; 4-month follow up in Trudeau, 2000).

In two instances (Jong et al., 2012, 2014), inadequate reporting made it impossible to calculate effect sizes, yet these numbers appear in other reviews (Jong et al., 2021; Kendrick et al., 2017). In these instances, we chose to use the means, standard deviations, and number of participants reported in Kendrick et al. (2016) under the assumption that they had the correct data from their communication with the researchers responsible for the studies.

### Quality Assessment / Assessment of Risk of Bias

Risk of bias was assessed independently by two authors or research assistants and discussions were subsequently held until a consensus was reached. We used the Cochrane Collaboration’s Risk of Bias Tool to assess risk in the following domains: (1) Random sequence allocation, (2) Allocation concealment, (3) Blinding of participants and therapists, (4) Blinding of outcome assessors, (5) Incomplete data, (6) Selective reporting and (7) Other bias. Risk of bias (RoB) is considered in separate domains as *high* (seriously weakened confidence in the effect estimate), *low* (unlikely to seriously alter the effect estimate), or *unclear*.

### Statistical Analyses

We calculated effect sizes for all outcome measurements where reporting allowed for it. Where possible, post-treatment measurement means, standard deviation, and number of respondents were used to calculate Cohen’s *d* and variance of this effect size. Alternatively, reported effect sizes were converted into Cohen’s *d*.

To account for the multiple effect sizes within studies, a three-level procedure was used in accordance with the procedure described in Assink and Wibbelink (2016). There were three levels of random variation in the analysis: between studies, between effects within studies, and between participants for each outcome measure.

All moderators were treated as categorical, and the overall effect was calculated for each category as well as differences in effects between the categories. The moderators included automatic guidance based on feedback scores (e.g., clinical support tool), child/adolescent or adult population, type/brand of MFS (OQ, PCOMS, or others), context (inpatient, outpatient, school, university counseling), length/dosage of treatment (short vs. long), and recipient of feedback (therapist or therapist and patient). Treatment was considered short if there were fewer than six sessions and the treatment lasted less than three months. We also calculated moderation of effect estimates from different outcome measures (symptoms/mental health outcomes, quality of life/functioning, and therapeutic alliance outcomes).

Effect sizes for the subgroup “not-on-track” (NOT) patients were taken out of the overall effect estimates, as this subgroup is theoretically assumed to have a greater effect than general patient populations. A separate effect estimate was produced in the same three-level structure that was the model for the main analyses.

For main analyses, a Shiny app (Chang et al., 2020) was developed for the procedure described by Assink and Wibbelink (2016). R code for the Shiny app is available in the GitHub repository: https://github.com/ToreWentzel-Larsen/threelevel

## Results

### Study Selection

**Fig. 1:**
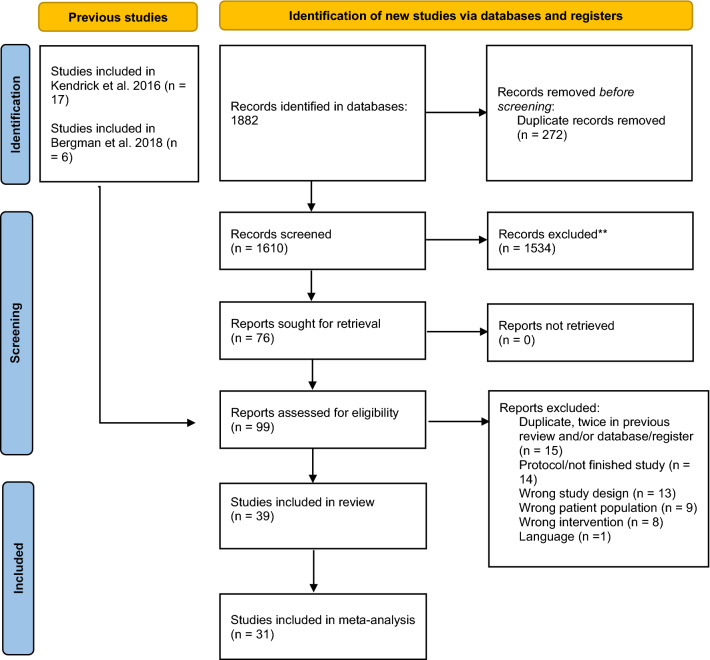
PRISMA flow diagram

Figure 1 presents a flow diagram of the search and inclusion process (PRISMA flow diagram). Fifteen of seventeen studies from Kendrick et al. (2016) were included. One study (Mathias et al., 1994/Mazonson et al., 1996) was excluded as we interpreted it to merely test a one-time screening and not a continuous outcome measurement and feedback system. One study (Berking et al., 2006) was excluded due to our research team’s limited mastery of the German language. All six studies from Bergman et al. (2018) were included.

New searches were done to update the findings from Kendrick et al. (2016) and Bergman et al. (2018). The searches resulted in 1882 abstracts, and 272 duplicates removed before the remaining 1610 articles’ titles and abstracts were screened. Two reviewers independently screened the abstract and title of each article, and conflicts were discussed to reach an agreement. Seventy-six studies were eligible for full-text screening; these were also reviewed by two authors independently.

Ten preregistered studies/protocols were identified, and corresponding authors were contacted and asked whether they had gone through with the study and had data to share. Two replied by sending articles in print or pre-print, which were included in the review (Bastiaansen et al., 2020; Bovendeerd et al., 2021).

The final sample combining relevant articles from Kendrick et al. (2016), Bergman et al. (2018), and the literature searches consisted of 39 studies. Appendix 1 provides an overview of the included studies. Appendix 2 provides an overview of ongoing studies based on protocols and preregistered studies not included in the current review.

### Study Characteristics

Thirty-nine studies were included in the review. Sample sizes ranged from 47 to 2884 participants. Most studies (22) took place in the United States or Europe (16) and studied effects of either the Outcome Questionnaire (OQ; 13 studies) or the Partners for Change Outcome Management System (PCOMS; 12 studies). The MFSs were mainly implemented in outpatient clinics (24 studies) or university/college counseling services (7 studies).

There were 31 studies that reported data allowing for the calculation of at least one effect estimate. In 10 studies, this could be done for one outcome measure, while 21 studies provided multiple quantitative outcome measures. Eight studies were excluded from the meta-analysis as we were unable to obtain quantitative post-treatment means and standard deviations or effect sizes that could be transformed to Cohen’s *d,* either in the article or via e-mail.

There was substantial heterogeneity in initiatives categorized as MFS, which ranged from systems that provided some initial measures at the beginning of therapy to extensive reports with graphical presentations throughout the treatment period. Self-reports are ubiquitous, but within children and youth initiatives, it is also possible to have reports from parents, therapists, and teachers. Ogles et al. (2006) only reported parent ratings as outcome measures although they also included children in interviews and thus likely had self-report data in the feedback reports. There was also heterogeneity in the dosage of therapy provided: one study had a mean of 1.7 sessions over a few days (Lester, 2012) while other studies had average treatment periods of longer than 6 months with an average of 40 sessions per patient (Lutz et al., 2015).

### Risk of Bias in Identified Studies

**Fig. 2:**
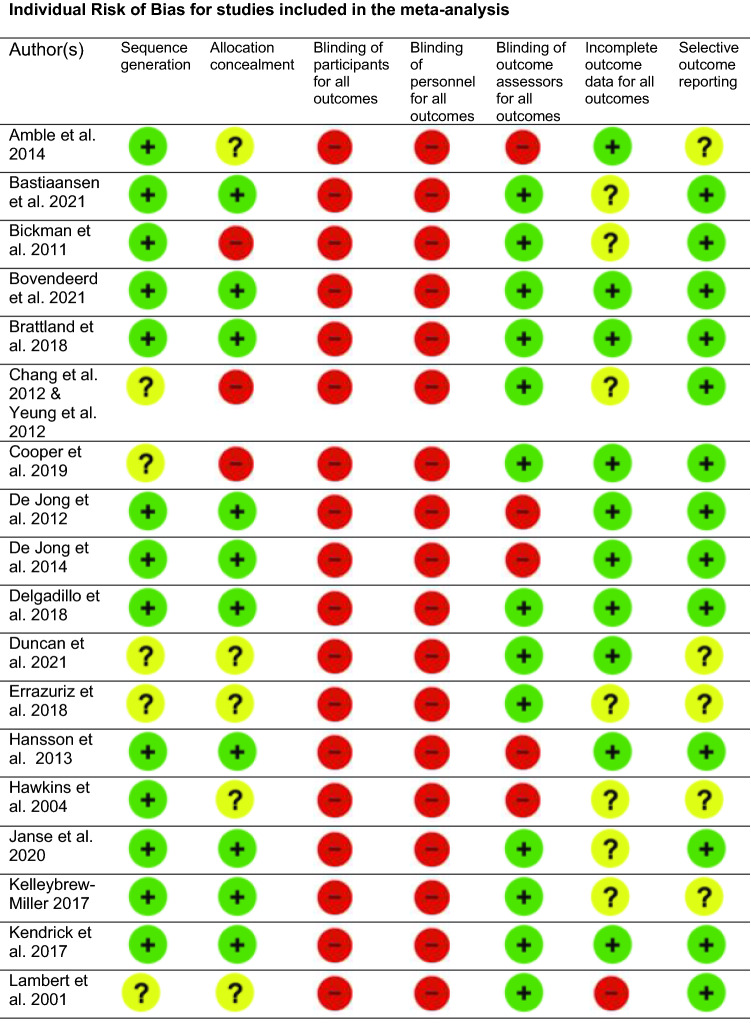

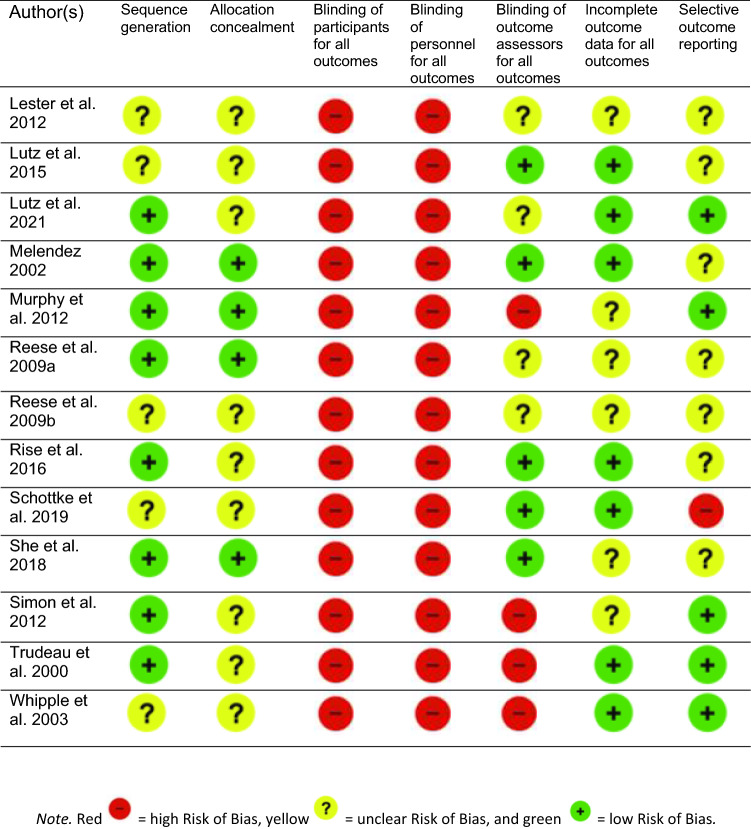
Cochrane Risk of Bias Comparison. Individual Risk of Bias for studies included in the meta-analysis.

**Fig. 3:**
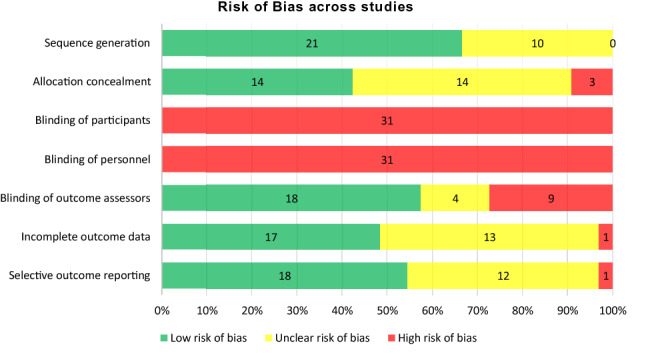
Risk of Bias summarized across studies

Risk of bias within and across the studies included in the meta-analysis is illustrated in Figs. 2, 3.

### Synthesis of Results (Meta-Analysis)

In the overall effect size estimate, we included all effect sizes that indicated a treatment outcome, either in symptoms/mental health or quality of life/functioning, and excluded alliance effect sizes. From the 30 studies included in the main meta-analysis, 67 effect sizes of treatment outcomes could be calculated based on a total sample of 13,807 participants. Of the studies included in the meta-analysis, most were conducted in outpatient clinics (18) or university counseling centers (7), while the others involved physicians/general practitioners (Chang et al., 2012; Kendrick et al., 2017), inpatient clinics (Lester, 2012) or a combination of inpatients and outpatients (Amble et al., 2015). There were 17 effect sizes from 13 studies that represented only “not on track” patients and were thus excluded from the main overall effect analysis.

The meta-analysis for clinical outcomes for full patient populations found a significant overall effect on treatment outcomes (*d* = 0.14, 95% CI [0.082–0.206], *p* < 0.001) favoring MFS over treatment as usual.

### Heterogeneity and Risk of Reporting Bias

A modified Egger test (Egger et al., 1997; Marengo & Montag, 2020) showed significant heterogeneity among the effect sizes comparing MFS to TAU (*Q* = 113.327, *df* = 65, *p* < 0.001). The likelihood ratio test showed that significant variance was present on the between-study level (SE = 0.12, p < 0.001), suggesting that between-study characteristics may impact the overall effect estimate.

There may be several reasons for heterogeneity, including both clinical and methodological differences and publication bias. In support of the last explanation, it should be noted that even among the identified articles, about one-fifth of the studies did not report outcomes in a way that made it possible to calculate effect sizes.

**Fig. 4:**
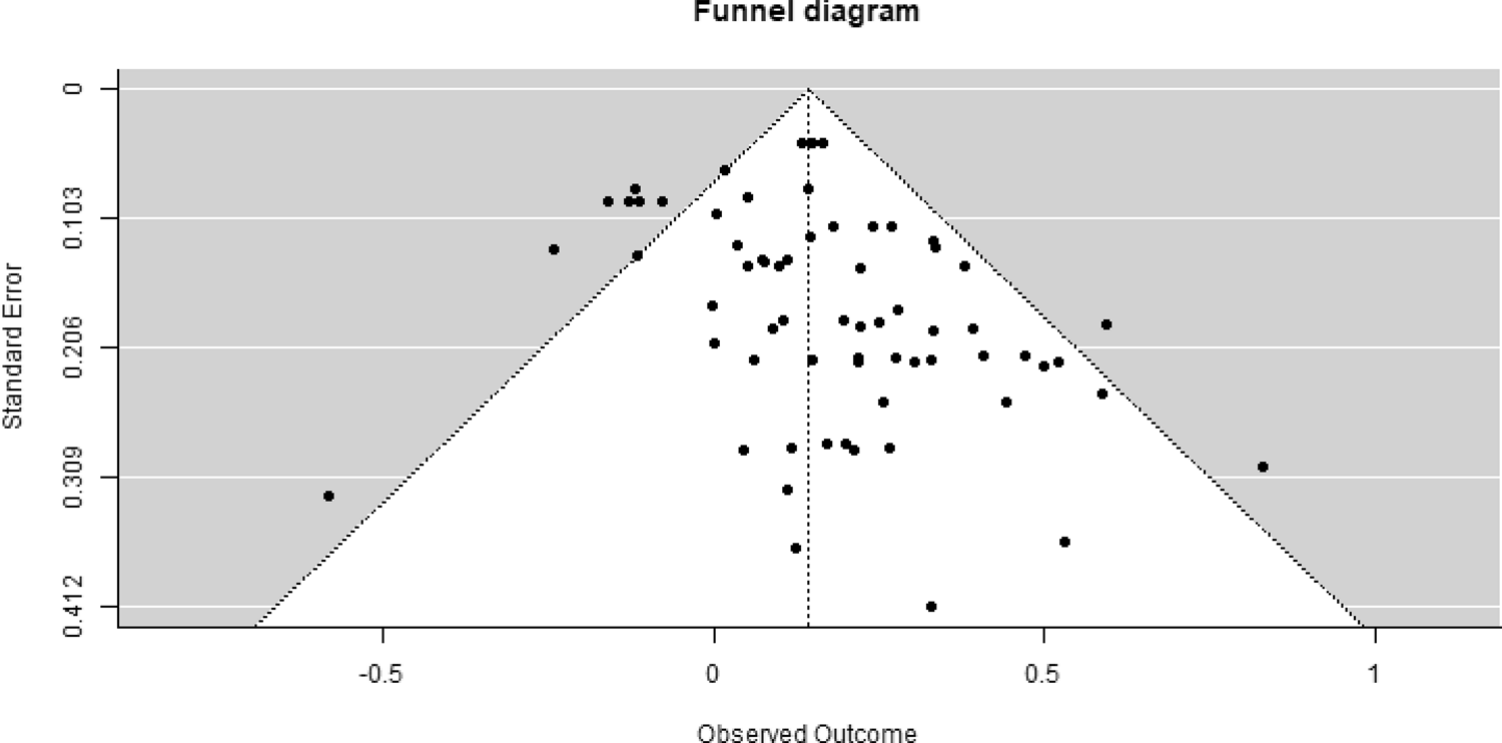
Funnel diagram of effect sizes from clinical outcome measures (symptoms and functioning/quality of life)

To test whether clinical or methodological differences could explain the asymmetric funnel diagram, we split the data into studies of PCOMS, OQ, and other systems. This resulted in more symmetric funnel diagrams for effect sizes in PCOMS studies and effect sizes in OQ studies. In both these cases (PCOMS studies only and OQ studies only), the modified Egger test was non-significant, implying that heterogeneity may not have been a problem when considering the different systems by themselves and indicating that the observed heterogeneity in the full sample might be due to clinical or methodological differences in the studies. Considering only the remaining studies (not PCOMS or OQ), the modified Egger test again showed significant heterogeneity (Fig. 4).

### Moderator Analysis

#### Type of Outcome

We found larger effects in outcome measures of quality of life or functioning (*d* = 0.24 CI [0.15 – 0.34], p < 0.001) and in alliance measures (*d* = 0.28 CI [0.03 – 0.53], *p* = 0.031) than in symptom or mental health measures (*d* = 0.11 CI [0.05 – 0.17], *p* < 0.001). Only the difference between symptom measures and quality of life/functioning was significant (difference = 0.101, CI [0.01 – 0.20] *p* = 0.046).

#### Not on Track

Thirteen studies provided effect sizes for NOT patients separately. The same three-level model was applied and an overall effect size estimate of Cohen’s *d* = 0.29 (CI [0.11 – 0.46], *p* = 0.003) was produced based on 17 effect sizes for treatment outcomes from these studies.

#### Length of Therapy

Length and dosage of therapy were tested as a moderator of the effect from MFS. Studies with shorter therapies attended to report a larger effect (*d* = 0.24, CI [0.11 – 0.38], *p* < 0.001) from MFS than studies with therapies of more than five sessions or longer than three months (d = 0.09, CI [0.01 – 0.17], p = 0.025). The difference was non-significant (difference = 0.15, CI [-0.01 – 0.31], *p* = 0.062).

#### Type of MFS

We tested type of MFS as a moderator for effects on symptoms and functioning. Three categories were coded: Partners for Change Outcome Management System (PCOMS), Outcome Questionnaire (OQ) and “Other.” A larger effect was found in PCOMS studies (*d* = 0.23, CI [0.13 – 0.34], *p* < 0.001) than in studies using OQ (*d* = 0.08, CI [-0.03 – 0.18], *p* = 0.149) and other systems (*d* = 0.12, CI [0.02 – 0.22], *p* = 0.021). Only the difference between effects in PCOMS studies and OQ studies was significant (difference = 0.16, CI [0.01 – 0.31], *p* = 0.04).

#### CST

We planned to test whether effects were moderated by the addition of CSTs or a similar decision support system to the MFS. Only two of the studies with adequate outcome data were identified as having such systems (Lutz et al., 2021; Whipple et al., 2003) and no significant moderator effect was found.

#### Context

Of the 67 effect sizes on treatment outcomes (symptoms or functioning/quality of life) from the 30 studies in the analysis, nearly all came from outpatient clinics or university counseling. We did not find significant moderation of effects from the context in which the MFS was implemented.

#### Adult or Youth Population

Only four of the studies in child and adolescent populations allowed for the calculation of effect sizes from the articles (Bickman et al., 2011; Cooper et al., 2021; Lester, 2012; Melendez, 2002). Age of the treated population significantly moderated MFS effects, and the tendency was for MFS to produce larger effects in child and adolescent studies (*d* = 0.29, CI [0.13 – 0.46], *p* < 0.001) than in studies of adult populations *(d* = 0.12, CI [0.06 – 0.18], *p* < 0.001). The difference was significant at a level of *p* < 0.05 (difference = 0.179, CI [0.00 – 0.36, *p* = 0.049).

### Recipient of Feedback

Studies varied in whether they presented the feedback data to the therapist only (21 studies) or to the therapist and patient (8 studies). The difference in effect from the MFS was non-significant when using the recipient(s) as a moderator (difference = -0.087 CI [0.067 – 0.222], *p* = 0.187).

#### Post-hoc Analysis

Significant moderation of effects was found when considering both different outcome measures and different types of MFS. MFS had a higher effect on measures of quality of life or functioning than on measures of symptoms of mental health problems. Furthermore, PCOMS studies had a larger effect than those investigating OQ or other systems. In both cases, we end up with a third variable problem as PCOMS studies frequently report quality of life/functioning through the Outcome Rating Scale (ORS). To investigate this, we performed a post hoc analysis with only PCOMS studies using the type of outcome measure as a moderator. In this analysis of a limited sample (k = 11, number of effect sizes = 27), results favored the feedback/PCOMS condition. Effects were significant in all types of outcomes, but larger in quality of life/functioning (Cohen’s *d* = 0.34, *p* < 0.001) and alliance measures (Cohen’s *d* = 0.32 *p* = 0.015) than in the mental health/symptoms measures (Cohen’s *d* = 0.11, *p* = 0.043). The difference between effects measured on quality of life/functioning and symptom scales was significant (difference = 0.23, CI [0.07 – 0.40], *p* = 0.008).

## Discussion

We found a significant small overall effect (Cohen’s *d* = 0.14) favoring MFS-assisted therapy compared to treatment as usual. Finding a small overall effect estimate on the general patient population in treatment for common mental health disorders can be considered in line with previous meta-analyses that found either non-significant differences (e.g., Kendrick et al., 2016) or significant small effects (e.g., De Jong et al., 2021; Knaup et al., 2009; Lambert et al., 2001; Shimokawa et al., 2010).

The current review reflects a substantial increase in the number of studies published since Kendrick et al. (2016) and Bergman et al. (2018). Thirty-nine studies were found, 31 of which had reporting that allowed for the calculation of 83 effect sizes for clinical outcome or therapeutic alliance. In Kendrick et al. (2017), 12 studies were included in the meta-analysis, and in Bergman et al. (2018), no meta-analysis could be conducted. Hence, the current review offers greater certainty compared to previous reviews. Still, some of the identified evidence is of low quality, as indicated by the RoB in the current review. Thus, findings must still be interpreted with some caution.

The relatively small overall effects of MFS implementation can be interpreted in multiple ways. One possibility is that this result reflects the potential MFSs have to impact treatment effects. Although of limited size, the overall estimate of effect is significant in our analysis, and a larger effect was found among patients who are normally expected to be regarded as treatment failures. Identifying and helping this group may be of clinical significance, and the costs of implementing and maintaining MFSs must be weighed against these possible effects. Others have found MFS to improve cost-effectiveness at a modest incremental cost to health services (Delgadillo et al., 2021). It is also worth noting that an MFS should work well in combination with other evidence-based treatments and MFS effects can thus add to, rather than replace, the effects of other efforts.

Alternatively, effect estimates might be affected by a lack of system adoption. Several studies show limited use of MFS when implemented (e.g., Bovendeerd et al., 2021; de Jong et al., 2012; Harris, 2011), and therapists report underuse due to both philosophical reasons and practical implementation barriers (Chung & Buchanan, 2019; Cooper et al., 2021). Clinicians report feeling intimidated by these systems (Lambert et al., 2019), and that these systems could be time-consuming and intrusive to clinical practice (Gelkopf et al., 2022). Future studies should both seek to overcome such barriers and follow the recommendation from Bickman et al. (2016) to calculate and report an implementation index to provide an estimate of the rate of adoption of such systems.

The identified studies also have considerable heterogeneity in features of the systems, and in the context and clinical practices in which they are used. The overall estimate is thus a statistical synthesis of potentially quite different MFSs. The moderator analyses give indications that some types of MFS are more beneficial for the patients than others. PCOMS studies find larger effect sizes than the others, but it is unclear whether this is confounded by a third variable. Also, youth populations seem to benefit slightly more than adult populations. Future studies should aim to provide answers to when and how MFS can cause a positive effect on treatment outcomes. A next step in the field could be to unpack the MFS initiatives and both statistically and experimentally test the impact of the components of these efforts (Leijten et al., 2021).

A more substantial overall effect was found in studies considering NOT patients, albeit from a smaller number of studies. Across 13 studies and 17 effect sizes, an overall effect size estimate of Cohen’s *d* = 0.29 was found. This is theoretically sound, as the feedback should function as a warning, and an impetus to change, in therapy that is not having the desired outcomes (Kluger & DeNisi, 1996; Scheier & Carver, 2003). The findings echo results from other reviews where larger effects were found in the NOT population (Kendrick et al., 2017; Shimokawa et al., 2010). On-track patients are less likely to have as much use for feedback, thus diluting the effect sizes of MFS initiatives in full patient populations.

A previous meta-analysis of OQ and PCOMS studies (Østergård et al., 2020) was criticized for including several very short treatments as this might limit the success of MFS (Duncan & Sparks, 2020). Consequently, we added duration as a moderator. Interestingly, we found that the effect sizes favored MFS conditions in both long and short therapies, with a tendency for MFSs to have larger effects in shorter treatments. Post hoc, one might speculate that patients in long and intensive treatment programs will often reach a certain level of familiarity with their therapist, and the therapist will have extensive knowledge about the patient, gradually making MFS obsolete. On the other hand, for patients in relatively short treatment programs, MFS may provide a way of building a better patient-therapist alliance and understanding in a shorter time span and fewer sessions.

## Limitations

The current study provides the most comprehensive evaluation of RCTs of MFSs in the treatment of common mental health disorders. Still, several limitations should be noted.

Although this study found a substantially higher number of studies than previous similar reviews, the number of studies in the meta-analysis is still limited, especially considering the heterogeneity in contexts, MFS functionality, and outcome measures.

Moderator analyses in the current review were in all cases based on limited samples in each category, and conclusions must therefore be regarded as highly uncertain. For instance, CSTs were reported as present in only three studies. In the studies within child and adolescent populations, in particular, there is an unfortunate combination of a very limited number of studies; substantial heterogeneity in context, MFS functionality, outcome measures, and population; and haphazard reporting on outcome measures. Only four child and adolescent studies could produce effect sizes, and two of these studies were pilot studies with very limited sample sizes.

Studies identified for the review came from 10 different countries but only three of the 39 included studies came from outside the United States and Europe. As noted by She et al. (2018), different cultures may have different traditions when it come to the therapist-client relationship, which will thus be affected differently by the use of MFSs.

The current review was limited to studies including patients with common mental health disorders and excluded studies with more than 10% of the sample in therapy for drug/alcohol treatment, dementia, learning disorders, psychosis, or eating disorders. MFSs might have different effects in these patient groups as they might face different challenges when it comes to self-reporting and as different information might be more relevant clinically. We also excluded group therapy studies, hence narrowing the review. The feedback loop might be different in group therapy in terms of how the therapist can address alliance ruptures and individual progress or change the course of therapy to accommodate individual feedback. Still, it would be of interest to see reviews of MFSs in these therapy forms and patient groups.

Risk of bias (RoB) evaluations of the individual studies indicate data of varying, and often low, quality. Future studies should ensure that trials are preregistered, clearly report outcome data (means, standard deviations, n) for the conditions separately, and be attentive to reporting and measures that might rule out alternative explanations for findings.

## Conclusions and Implications for Research

The current findings indicate that there is a small positive effect on clinical outcomesof common mental health problems by introducing an MFS to assist treatment. Further studies should investigate when and how these effects are produced and in what type of outcomes we can expect to find them.

A problem with many of the identified studies is that they used the same measure in the MFS as they use as an outcome measure. This might lead to an inflation of effect estimates, a concern raised by other reviewers (e.g., Østergård et al., 2020). In interpreting findings from these studies, we face several challenges as therapy might be focused too narrowly on the issues addressed in the MFS surveys or problems with response set or social desirability biases. In our data, effects were largest in PCOMS studies and when the outcome measure used was the Outcome Rating Scale (ORS), which also appears as half of the survey in the MFS. To address this issue, we would strongly encourage future research to include independent outcome measures.

Another source of complication in interpreting the meta-analysis is our suspicion that the reported effect estimates are quite heavily influenced by poor implementation and limited utilization of the feedback. The studies that reported any kind of data on MFS implementation or therapists’ use of feedback often provided data that indicate low therapist adoption of the systems, and two indicated larger effects as implementation becomes more successful (Bickman et al., 2016; Brattland et al., 2018). Future studies should follow Bickman et al.’s (2016) suggestion to, at a minimum, report an implementation index reflecting the extent to which surveys are answered by patients and feedback is viewed by therapists. We also need more knowledge about the contextual elements needed for MFSs to be successful.

## Supplementary Information

Below is the link to the electronic supplementary material.

Supplementary file1 (PDF 46 KB)

Supplementary file2 (PDF 77 KB)

## Data Availability

Data files are available in the GitHub repository: https://github.com/kRognstad/MFS-meta or by contacting the corresponding author. R code for the Shiny app is available in the GitHub repository: https://github.com/ToreWentzel-Larsen/threelevel
