# Supplementary material for: A Systematic Review and Meta-Analysis of Measurement Feedback Systems in Treatment for Common Mental Health Disorders
Source: Adm Policy Ment Health. 2022 Nov 25;50(2):269–82. doi: 10.1007/s10488-022-01236-9 (PMC9931854; doi:10.1007/s10488-022-01236-9)
Supplement: Supplementary file 1 — Supplementary file1 (PDF 46 KB) [file 10488_2022_1236_MOESM1_ESM.pdf]

Study (publication)	Sample size (randomized)	Country	Population	Type of MFS	Treatment length/dosage (months/average no. of sessions)	Treatment context	Included in main meta-analysis	Included in NOT-analysis
Amble et al. 2014	302	Norway	Adult patients (mean age 35.8 years)	OQ-45	NA/9,9	Out- and inpatient clinics	Yes	No
Bastiaansen et al. 2021	106	the Netherlands	Adult patients (mean age 32.8) in depression treatment	ZELF-i	NA/NA	Outpatient clinic	Yes	No
Bickman et al. 2011	340	USA	Youths aged 11 to 18 (mean age 15)	Contextualized Feedback Systems (CFS)	3-6/11	Outpatient clinic/at-home-service	Yes	No
Bickman et al. 2016 (rural)	141	USA	Youth at least 11 years old (mean 14.6)	Contextualized Feedback Systems (CFS)	3-6/10	Outpatient clinic	No	No
Bickman et al. 2016 (urban)	116	USA	Youth at least 11 years old (mean 14.2)	Contextualized Feedback Systems (CFS)	3-6/11	Outpatient clinic	No	No
Bovendeerd et al. 2021	1933	the Netherlands	Adult patients (mean age 38.2) with mild to moderate psychological disorders	PCOMS	>6/7	Outpatient clinic	Yes	No
Brattland et al. 2018	170	Norway	Adult patients (mean age 34) who suffer from mental health problems of all diagnostic categories	PCOMS	NA/12,5	Outpatient clinic	Yes	No
Chang et al. 2012 and Yeung 2012	915	USA	Adult patients (mean age 46.0) with depression	Depression severity feedback	NA/NA	Primary care physician	Yes	No
Connolly & Gibbons 2015	100	USA	Adult patients (mean age 39) patients seeking services for depression	Community Clinician Feedback System (CCFS)	<3/6	Outpatient clinic	No	No
Cooper et al. 2021	38	United Kingdom	Children 5–11 years old (mean age 8.5)	PCOMS	3-6/12 to 36	School counseling	Yes	No
De Jong et al. 2012	544	the Netherlands	Adult patients (mean age 36.8) with a wide range of psychiatric disorders, including mood, anxiety, adjustment and personality disorders	OQ-45	NA/NA	Outpatient clinic	Yes	Yes

De Jong et al. 2014	399	the Netherlands	Adult patients (mean age 38.2) in psychotherapy	OQ-45	>6/32,3	Outpatient clinic	Yes	Yes
Delgadillo et al. 2018	2884	England	Adult patients (mean age 39.2) offered protocol-driven, evidence-based psychological interventions for depression and anxiety disorders	Patient Case Management Information System	NA/6	Outpatient clinic	Yes	Yes
Duncan et al. 2021	285	USA	Adult patients (mean age 37.9) receiving behavioral health services	PCOMS	NA/4,3	Outpatient clinic	Yes	No
Errazuriz et al. 2018	547	Chile	Adult patients (mean age 41.3) receiving individual psychotherapy	OQ-45	NA/7,8	Outpatient clinic	Yes	No
Garland et al. 2017	194	USA	Youth clients aged 4 to 17 (mean age 10.1) who received psychotherapy	OQ and Treatment Support Measure	<3/5	Outpatient clinic	No	No
Hansson et al. 2013	374	Sweden	Adult patients (mean age 39) with mental disorders with the exception of substance use disorders, schizophrenia and other psychotic disorders.	OQ-45	NA/15,8	Outpatient clinic	Yes	Yes
Harris et al. 2012	207	USA	Adult patients (mean age 36.1) seeking psychotherapy	OQ-45	NA/6,6	Outpatient clinic	No	Yes
Hawkins et al. 2004	201	USA	Adult patients (mean age 30.8) seeking outpatient psychotherapy	OQ-45	NA/8,2	Outpatient clinic	Yes	No
Janse et al 2020	368	the Netherlands	Adult patients (mean age 41.4) in secondary care	PCOMS	>6/15	Outpatient clinic	Yes	No
Kellybrew-Miller 2014	161	USA	Adult patients (mean age 36.4) with a wide range of mental health concerns	PCOMS	Minimum 2 sessions	Outpatient clinic	Yes	No
Kendrick et al. 2017	47	England	Adult patients (mean age 44) diagnosed with a new episode of depression.	No specified name – multiple scales administered	NA/NA	General practices	Yes	No
Lambert et al. 2001	609	USA	Adult clients (mean age 22.2) referred or self-referred for personal concerns	OQ-45	NA/3,6	University counseling center	Yes	Yes
Lester 2012	118	USA	Adolescents 12-18 years old (mean age 14.7) admitted for acute stabilization	PCOMS	<3/1,7	Inpatient	Yes	No

Lutz et al. 2015	349	Germany	Adult patients (mean age 44.8) in private practices	No specified name – multiple scales administered	NA/40,6	Outpatient clinic	Yes	No
Lutz et al. 2021	614	Germany	Adult patients (mean age 36.3) receiving cognitive behavioral therapy	Decision support system	>6/34,1	Outpatient clinic	Yes	No
McClintock et al. 2017	79	USA	Undergraduates at university (mean age 19.3) with depressive symptoms	Common Factors Feedback (CFF) system	<3/4,1	Psychotherapy laboratory (university)	No	No
Melendez 2002	69	USA	Youth between the ages of 12 and 18 with behavioral disorders	No specified name – Ohio Scales administered	<3/NA	Therapeutic day-school	Yes	No
Murphy et al. 2012	110	Ireland	Adult patients (mean age 23.8)	PCOMS	NA/3,7	University counseling service	Yes	No
Ogles et al 2006	72	USA	Youth (mean age 13.3) receiving wraparound services instituted through a family team	No specified name – multiple scales administered	NA/NA	Wraparound approach (family/home support)	No	No
Probst et al. 2013	209	Germany	Adult patients (mean age 47.6)	OQ-45	<3/NA	In-patient psychosomatic clinics	No	No
Reese et al. 2009 – Study 1	74	USA	Students (mean age 20.1) receiving individual therapy	PCOMS	NA/NA	University counselling service	Yes	No
Reese et al. 2009 – Study 2	74	USA	Students (mean age 32.9) receiving individual therapy	PCOMS	NA/6,9	Training clinic for a marriage and family therapy master's program (MFC)	Yes	Yes
Rise et al 2016	69	Norway	Adult patients (mean age 29.9) offered psychotherapy	PCOMS	<3/3,8	Outpatient clinic	Yes	No
Schottke et al. 2019	203	Germany	Adult patients (mean age 36.0) treated with cognitive behavioral therapy	Fragebogen zur Evaluation von Psychotherapie-verläufen	NA/30	Outpatient clinic	Yes	No

She et al. 2018	332	China	University students (mean age 21.4) with a variety of problems, e.g. interpersonal and family problems, emotional problems, self-injury, trauma-related symptoms.	PCOMS	NA/5,2	College counseling center	Yes	Yes
Simon et al. 2012	464	USA	Adult patients (mean age 36.1) seeking psychotherapy	OQ-45	NA/6,6	Outpatient clinic	Yes	Yes
Trudeau 2000	127	USA	Adult patients (mean age 33.9) in mental health therapy	OQ-45	NA/6,7	Outpatient clinic	Yes	No
Whipple et al. 2003	981	USA	University students (mean age 22.9) referred or self-referred for personal concerns	OQ-45	NA/NA	University counseling service	Yes	Yes
