# Supplementary material for: A Systematic Review and Meta-Analysis of Measurement Feedback Systems in Treatment for Common Mental Health Disorders
Source: Adm Policy Ment Health. 2022 Nov 25;50(2):269–82. doi: 10.1007/s10488-022-01236-9 (PMC9931854; doi:10.1007/s10488-022-01236-9)
Supplement: Supplementary file 2 — Supplementary file2 (PDF 77 KB) [file 10488_2022_1236_MOESM2_ESM.pdf]

Appendix 1. Ongoing studies

GET.FEEDBACK.GP multicenter randomized controlled trial<br>Study protocol: Lehmann, M., Kohlmann, S., Braunschneider, L-E., Marx, G., Eisele, M., Zapf, A., Scherer, M., & Löwe, B. (2021). Clinical effectiveness of patient-oriented depression feedback in primary care: The empirical method of the GET.FEEDBACK.GP multicenter randomized controlled trial. *Contemporary Clinical Trials, 110*, 106562
Community Study of Outcome Monitoring for Emotional Disorders in Teens (COMET).<br>Study protocol: Jensen-Doss, A., Ehrenreich-May, J., Nanda, M. M., Maxwell, C. A., LoCurto, J., Shaw, A. M., Souer, H., Rosenfield, D., & Ginsburg, G. S. (2018). Community Study of Outcome Monitoring for Emotional Disorders in Teens (COMET): A comparative effectiveness trial of a transdiagnostic treatment and a measurement feedback system. *Contemporary Clinical Trials, 74*, 18-24.
The missing link between daily life and the therapist's office<br>Registration: http://www.who.int/trialsearch/Trial2.aspx?TrialID=NTR7381<br>Study protocol: Riese, H, von Klipstein, L., Schoevers, R. A., van der Veen, D. C., & Servaas, M. N. (2021). Personalized ESM monitoring and feedback to support psychological treatment for depression: a pragmatic randomized controlled trial (Therap-i). *BMC Psychiatry, 21*, 143.
Doing More With Less: optimizing Psychotherapeutic Services in the Mental Health System<br>Registration: https://clinicaltrials.gov/show/NCT03608449
Optimization of therapy outcome through client feedback in cognitive behavioral therapy of children and adolescents with internalizing and externalizing disorders<br>Registration: http://www.who.int/trialsearch/Trial2.aspx?TrialID=DRKS00016737

Enhancing the Clinical Effectiveness of Depression Screening Using Patient-targeted Feedback in General Practices: the GET.FEEDBACK.GP Multicentre Randomized Controlled Trial<br>Registration: https://clinicaltrials.gov/show/NCT03988985
Tracking and Improving Trust/Respect<br>Registration: https://clinicaltrials.gov/show/NCT04392336
Assessing Psychotherapy Outcome With Feedback<br>Registration: https://clinicaltrials.gov/show/NCT02023736
Routine Outcome Monitoring in Mental Health Outpatient (ROM-Shalvata)<br>Registration: https://clinicaltrials.gov/ct2/show/NCT02095457
